# Supplementary figures and images for: Insights into the origin, hybridisation and adaptation of Candida metapsilosis hybrid pathogens
Source: PLoS Pathog. 2025 Jan 17;21(1):e1012864. doi: 10.1371/journal.ppat.1012864 (PMC11781744; doi:10.1371/journal.ppat.1012864)

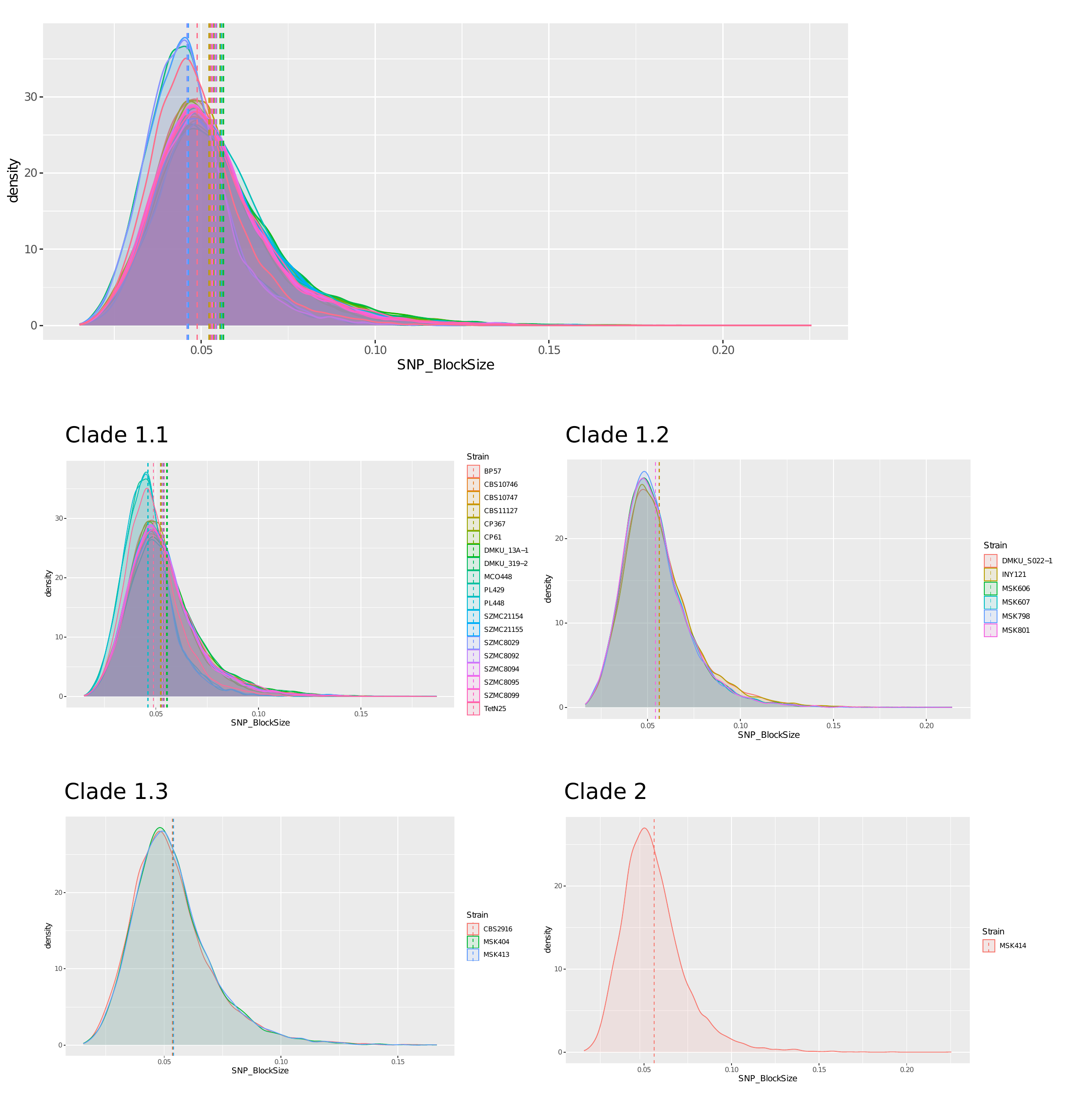

Supplement: S1 Fig — Plot of the density of sequence divergence in heterozygous blocks (larger than 100 base pairs) of all C. metapsilosis analysed in this study. (TIF) [file ppat.1012864.s004.tif]

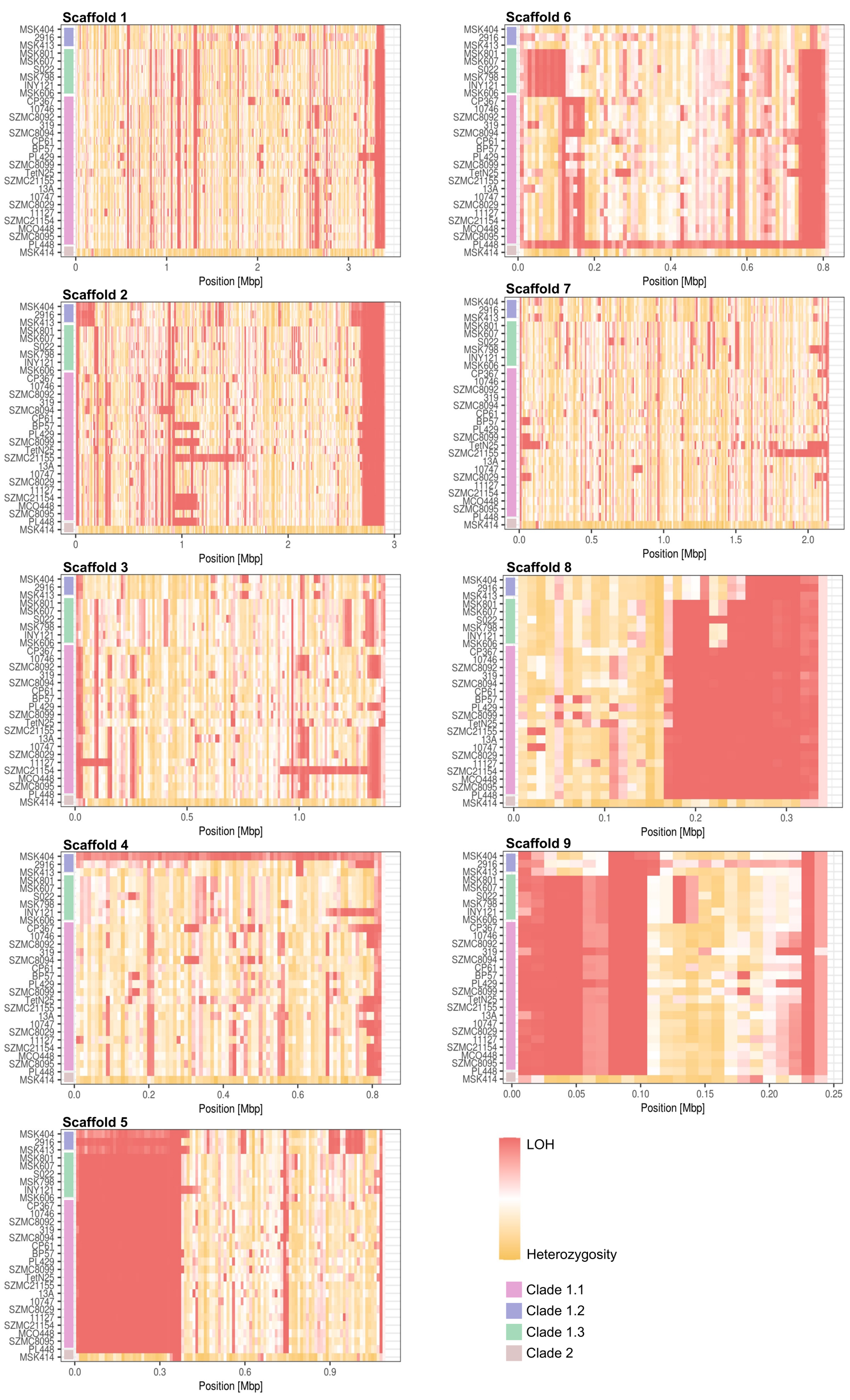

Supplement: S2 Fig — JLOH—cluster module was first used to study the similarity between LOH blocks and assess clustering between samples. The tool recapitulated the four clusters using a maximum distance parameter of 0.35 which means that the items in a cluster (LOH blocks) must share at least 65% identity. (TIF) [file ppat.1012864.s005.tif]

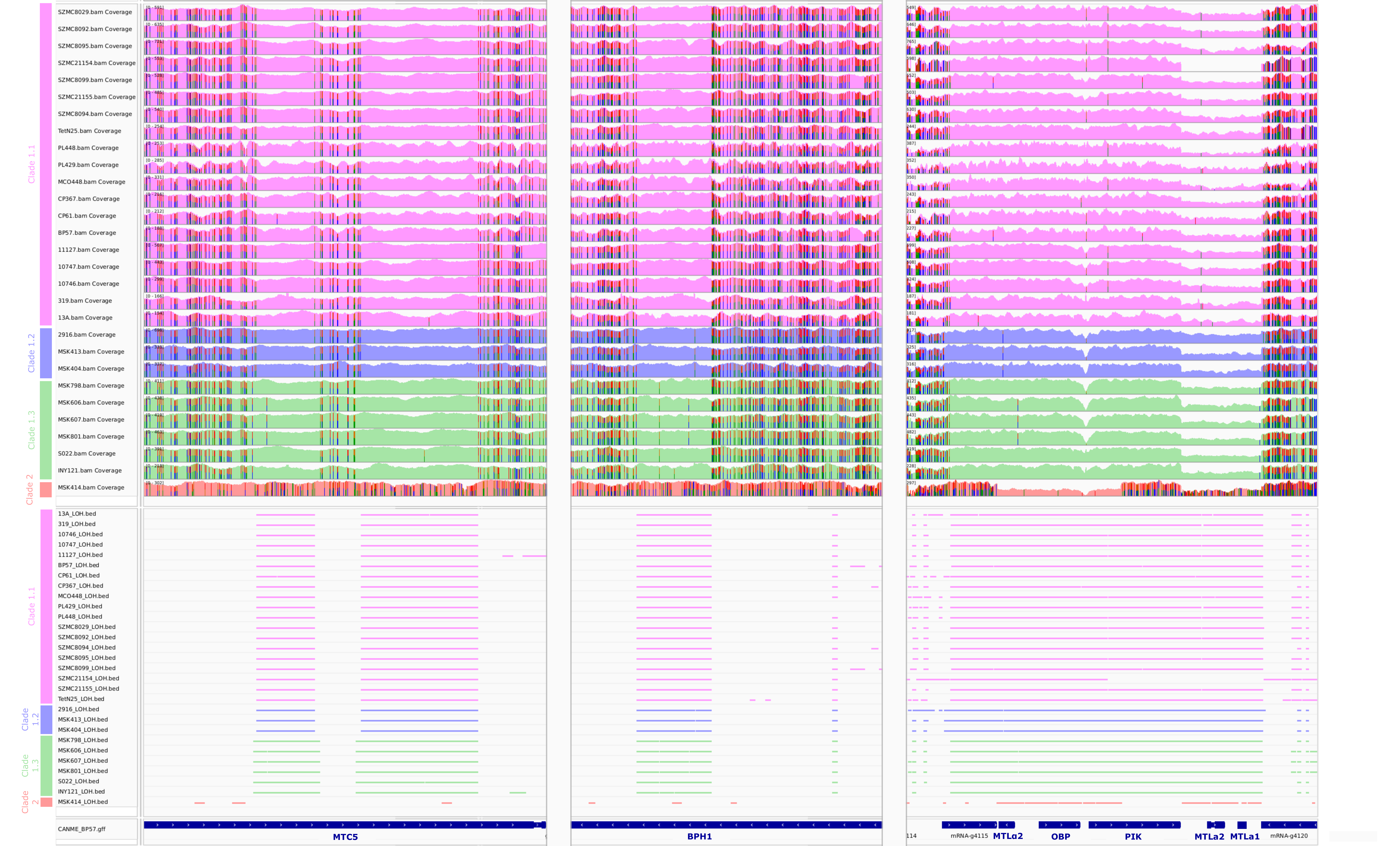

Supplement: S3 Fig — Graph showing IGV coverage tracks (top) and LOH blocks (bottom) of studied strains. SNPs are shown as vertical lines on the tracks. The three vertical panels show, from left to right, genes MTC5, BPH1 and the MTL locus. (TIF) [file ppat.1012864.s006.tif]

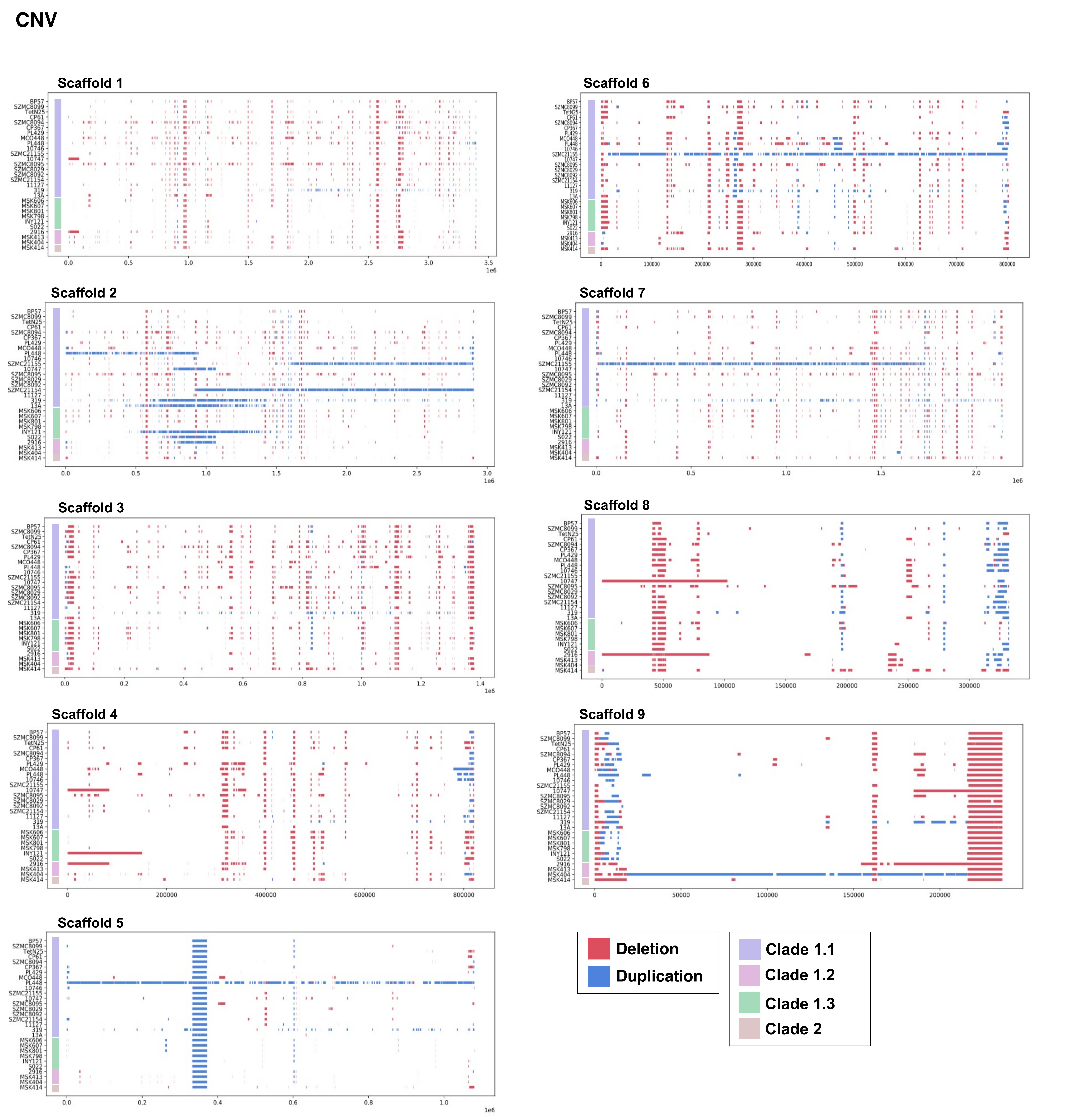

Supplement: S4 Fig — Graphs showing the presence of deletions (red) and duplications (blue) across the genome of C. metapsilosis hybrid strains. (TIF) [file ppat.1012864.s007.tif]

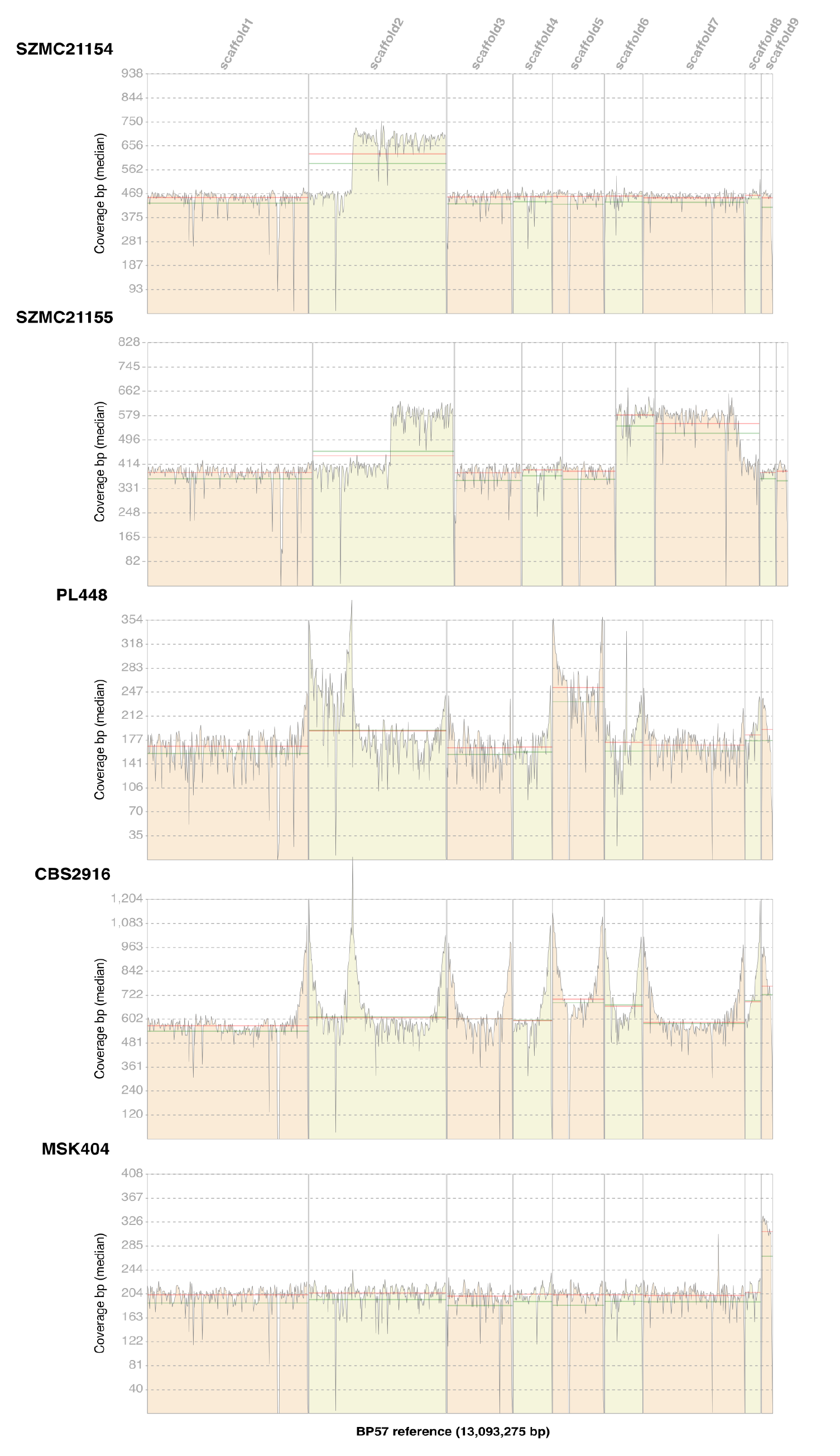

Supplement: S5 Fig — Graphs showing depth of coverage across the genome of C. metapsilosis samples. Scaffold boundaries are marked by vertical lines on the graphs. Horizontal red lines mark the median depth of each scaffold while the horizontal green lines refer to the average depth of coverage per scaffold. (TIF) [file ppat.1012864.s008.tif]

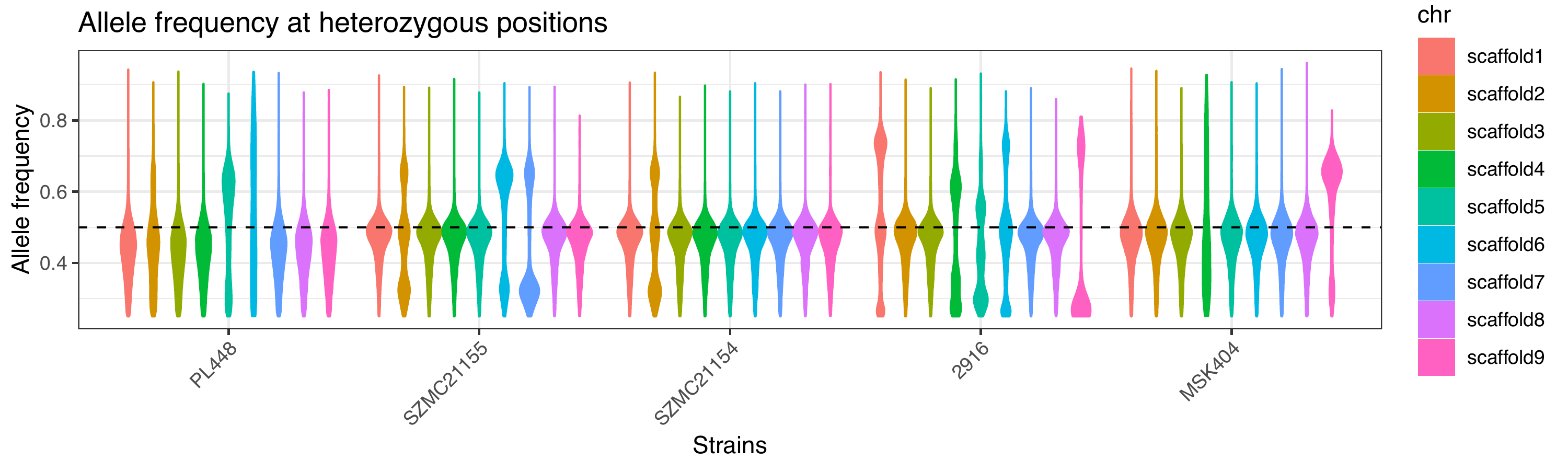

Supplement: S6 Fig — Graphs showing the allele frequency in heterozygous SNPs for each chromosome of the five C. metapsilosis aneuploid strains PL448, SZMC21155, SZMC21154, CBS2916 and MSK404. (TIF) [file ppat.1012864.s009.tif]

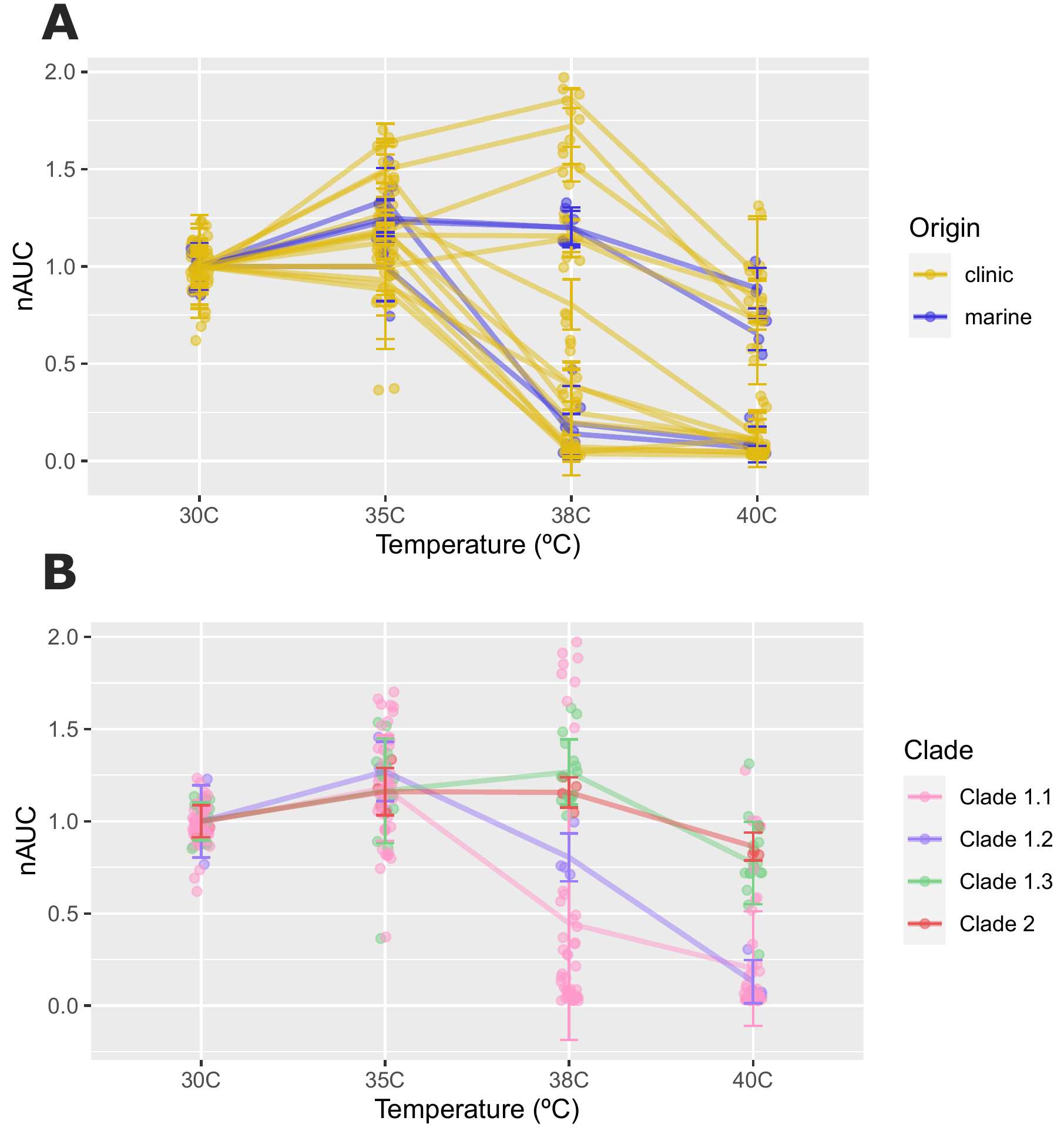

Supplement: S7 Fig — Growth measurements were taken every 15 minutes during a period of 24 hours in solid YPD rich medium. Four replicates per strain are included in this analysis. The growth of each strain at each temperature is relative to the average growth of the four replicates of that strain at 30°C. Strains are coloured by (A) origin of isolation or (B) strain name. (TIF) [file ppat.1012864.s010.tif]

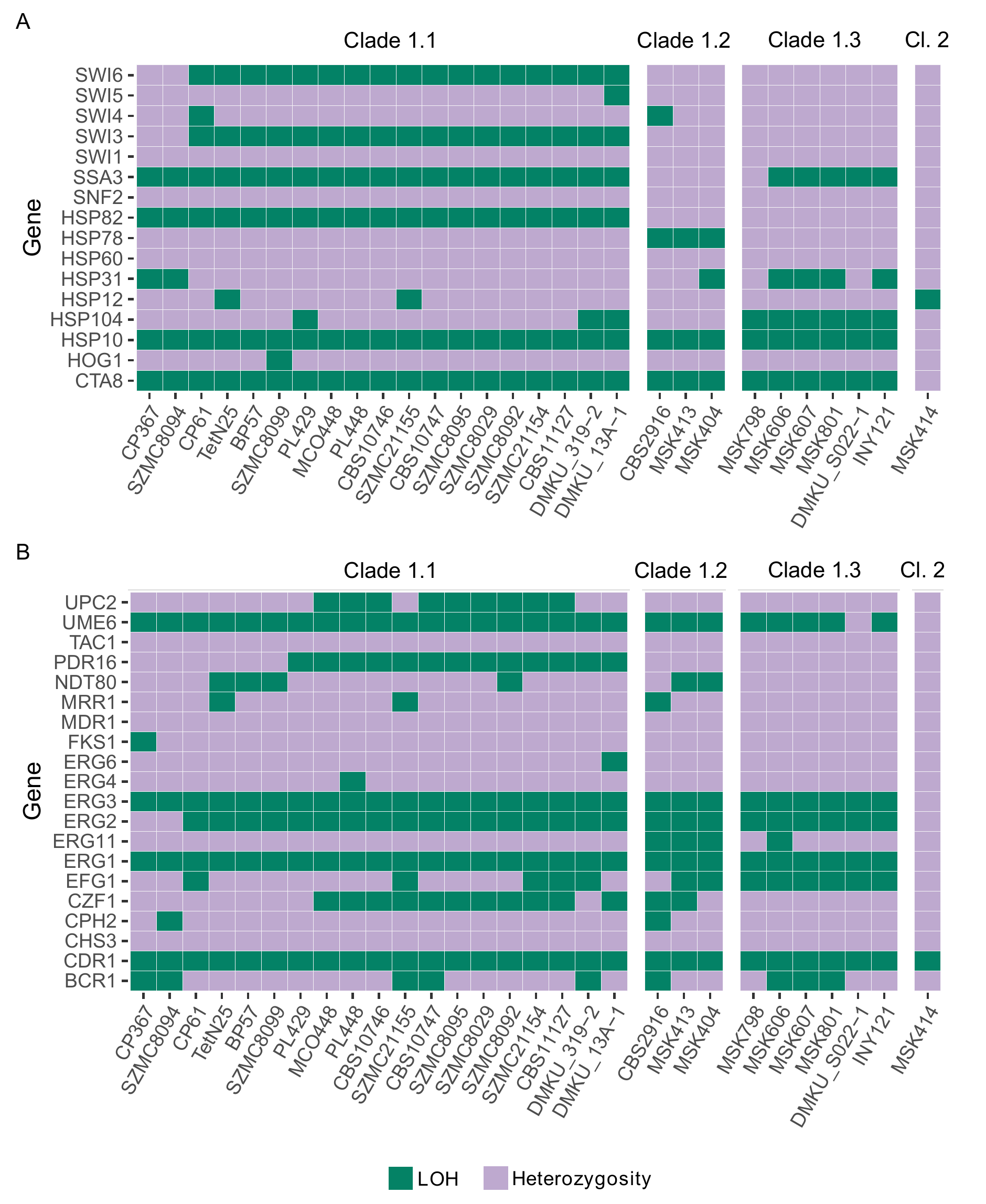

Supplement: S8 Fig — Inference of LOH (green) or heterozygosity (purple) in a subset of selected genes. More than 50% of the gene length must be covered by LOH in order to be categorised as such. (TIF) [file ppat.1012864.s011.tif]

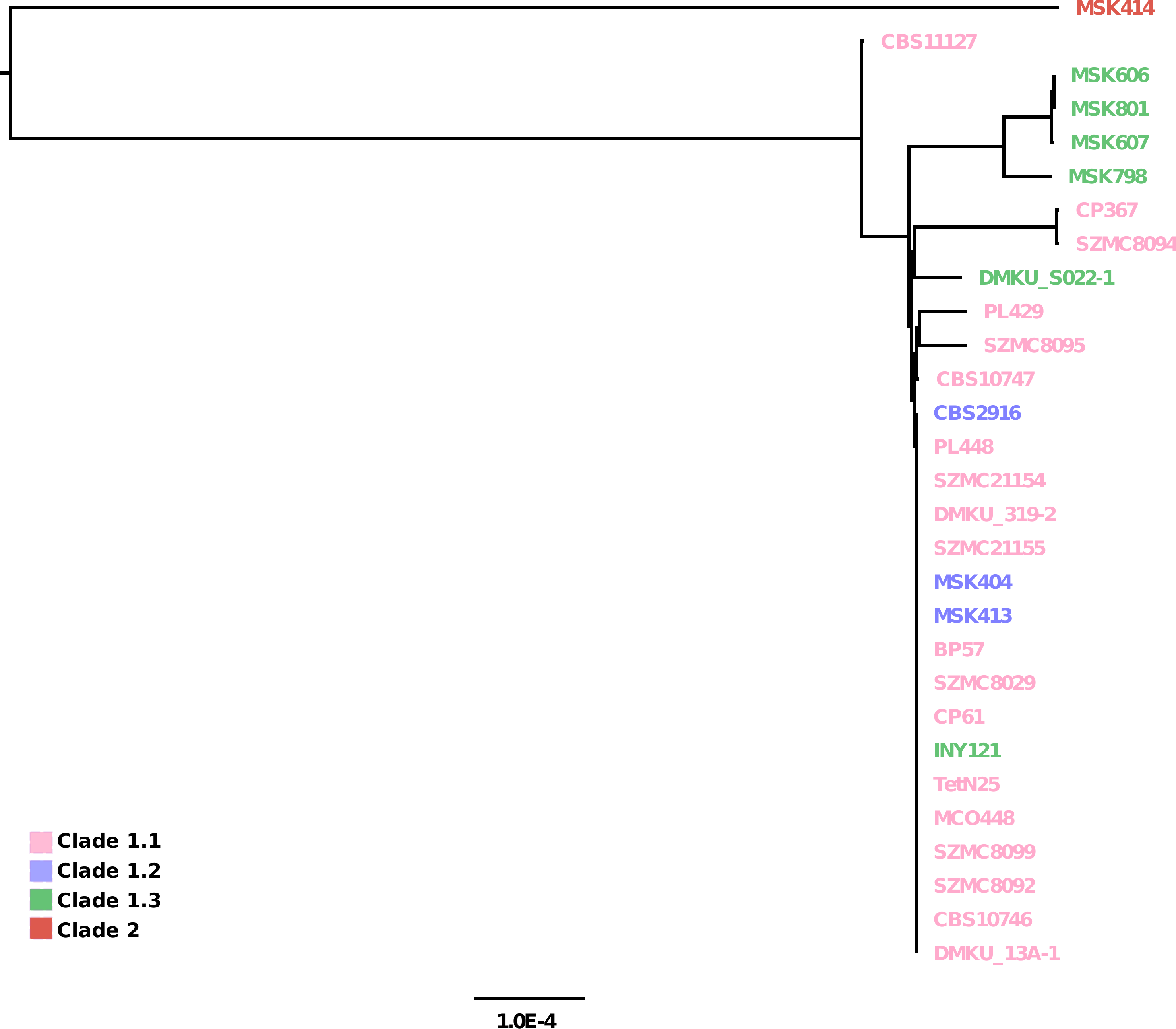

Supplement: S9 Fig — Maximum-likelihood tree based on variants of the mitochondrial genome of C. metapsilosis strains. Nuclear clades are marked in different colours. (TIF) [file ppat.1012864.s012.tif]
