# Supplementary material for: Insights into the origin, hybridisation and adaptation of Candida metapsilosis hybrid pathogens
Source: PLoS Pathog. 2025 Jan 17;21(1):e1012864. doi: 10.1371/journal.ppat.1012864 (PMC11781744; doi:10.1371/journal.ppat.1012864)
Supplement: Attachment — Submitted filename: Comments metapsilosis PLOS pathogens.pdf [file ppat.1012864.s014.pdf]

Dear Editor,

As you will see we have addressed all concerns. The main criticism seemed to be the distinction of a new hybridisation event. We have reconsidered this issue and agree with the reviewers that we do not have sufficient support for this claim. We have made all necessary changes. As for the requested additional experiments, we unfortunately are limited in the number of strains we have at our disposal, and much of the data comes from public repositories or from material shared in the form of DNA (not strains), we have therefore toned down some of the statements to make clear our conclusions are limited to the tested strains. I also want to apologize for the delay, as the first author has left the laboratory and it took us time to find someone to continue the work. We have also added a new co-author. We think the manuscript has substantially improved and we than you and the two reviewers for your help.

Best wishes, Toni

Reviewer's Responses to Questions

Part I - Summary
Please use this section to discuss strengths/weaknesses of study, novelty/significance, general execution and scholarship.

Reviewer #1: The manuscript reports genomic analysis of isolates of Candida metapsilosis, a pathogenic yeast that has received relatively little attention. The manuscript includes analysis of phylogeny and loss of heterozygosity (LOH), and phenotypic analysis of thermotolerance, drug resistance, and virulence in the Galleria model. It includes analysis of some of the first environmental isolates of C. metapsilosis (from marine waters). The study extends our understanding of this species. Interestingly, all the environmental isolates of C. metapsilosis were found to be hybrids. No isolates representing the pure parental lineages were discovered, unlike in the related hybrid species C. orthopsilosis for which both parents have been discovered.

Response: We thank the reviewer for the accurate account of our results and his/her appreciation for the interest of the topic.

Reviewer #2: Candida metapsilosis is a diploid fungal pathogen that has origins in hybridization, with the contributing parental genotypes not yet linked to current haploid species. In this work the authors target sequencing of five environmental isolates to characterize their contribution to the population structure and study loss of heterozyosity events linked to phenotypic differences.

Environmental reservoirs of Candida species are of high interest, and while the sampling is small, the genotypes recovered are diverse, present in one known and one newly described phylogenetic clade. Evidence for the third clade as a "new hybridsation event" is limited and this conclusion based on the data in Figure 1 seems overstated. Three assays are carried out to assess phenotypic diferences between the isolates; growth at high temperature, sensitivity to two antifungal drugs, and virulence in a galleria model. For both temperature

and drug sensitivity there are striking preliminary results of differences between isolates; the genome analysis finds candidate genes in LOH regions, however none are pursued to confirm a specific role linking genotype to phenotype. The galleria virulence work is even more preliminary in measuring a single isolate as a representative of each clade, so can not disentangle clade (genetic) from sample origin differences and correlate these measurements with other work. The conclusions contribute significantly to placing environmental isolates in the population structure of this species and describing phenotypic and genomic differences.

Response: We thank the reviewer for the interest in our findings. As both reviewers raised reasonable doubts, we have now re-evaluated our consideration for the third hybridisation event. As you will see we now conclude that there is not sufficient evidence for such a third hybridisation event, and instead we interpret the divergence as an early diversification into three subclades from a single hybridization event. Regarding the experimental confirmation of the candidate genes, unfortunately despite several attempts we have not managed to implement genetic manipulation in this species, and the literature indicates a high potential to induce off-target LOH in this group of species, which would make our results inconclusive. Finally, as we cannot repeat the experiment including a larger number of isolates per clade, as many of the genome data belongs from isolates from other laboratories, we have toned down our conclusions in this respect, acknowledging that the observed strain differences might not necessarily indicate clade-level differences.

Part II – Major Issues: Key Experiments Required for Acceptance
Please use this section to detail the key new experiments or modifications of existing experiments that should be <u>absolutely</u> required to validate study conclusions.

Generally, there should be no more than 3 such required experiments or major modifications for a "Major Revision" recommendation. If more than 3 experiments are necessary to validate the study conclusions, then you are encouraged to recommend "Reject".

Reviewer #1: My main comment concerns the section beginning on Line 143, “C. metapsilosis hybrids descend from at least four hybridization events between unknown parentals”. The four events are: one postulated hybridization between parents “A” and “C” (O’Brien et al 2022, ref. 26) and three postulated hybridizations between parents “A” and “B”. In my opinion, the evidence that there were three separate hybridizations between parents A and B is weak and this conclusion is not justified. Del Olmo et al confuse the situation by presenting an inaccurate summary of previous work, and also by renaming some clades, before presenting their own results.

The relevant background is:
- Previous work showed that almost all isolates of C. metapsilosis are “AB” hybrids, i.e. hybrids between two parental lineages that have been named A and B. One isolate, MSK414, is an “AC” hybrid, i.e. a hybrid between lineage A and another lineage C that is different from lineage B (O’Brien et al 2022, ref. 26). O’Brien et al concluded that two independent hybridization events occurred in the C. metapsilosis clade (an A/B hybridization and an A/C hybridization) and I agree.
- Mixao et al 2023 (ref. 28) constructed a phylogenetic tree of C. metapsilosis strains (their Fig. 3b). They designated two clades, Clade 2 (containing only MSK414, i.e. the AC hybrid) and Clade 1 (containing all other C. metapsilosis isolates, i.e. the AB hybrids). They also subdivided Clade 1 into two subclades called Clade 1.1 (containing most of the isolates) and

Clade 1.2 (containing only CBS2916 and MSK446).
- In the current manuscript, Del Olmo et al have renamed these clades, so that the former Clade 1.1 is renamed Clade 1, the former Clade 1.2 is renamed Clade 2, and they identified a new clade that they call Clade 3. MSK414's clade is renamed Clade 4. Del Olmo don't mention that they changed the clade names that were introduced by their own lab a year ago (Mixao et al 2023), so they need to do this.

Response: We now have revised the whole section, present better the past work and use names that are consistent with the mentioned previous publications.

The part of the manuscript that is an inaccurate summary begins on Lines 145-147. It says "So far, studies divide all C. metapsilosis strains except one [MSK414] into two main clades (named Clade 1 and Clade 2) reflecting two independent hybridization events between … A and B". It cites refs 20 (Pryszcz et al 2015), 26 (O'Brien et al 2022), and 28 (Mixao et al 2023). However, I cannot find any statements in any of these 3 papers saying that there were two independent A/B hybridizations. In fact, they all reached the *opposite* conclusion:
- Pryszcz et al (2015), which is from the authors' lab, said "All sequenced C. metapsilosis clinical isolates result from a single hybridization event" (page 6).
- Mixao et al (2023), which is also from the authors' lab, said "We consider that, with the current data at hand, a shared hybridization event followed by early separation of the two subclades [Clades 1.1 and 1.2] is the most parsimonious scenario…". They reached this conclusion after finding two blocks of LOH, >1 kb long, that were shared by the two subclades (page 6).
- O'Brien et al (2022) was about MSK414, and concluded (correctly) that there were separate A/B and A/C hybridizations. They described the A/B hybridization as "a single event" (page 7) and they did not make any claim that there was more than one A/B hybridization.
- Therefore, the claim (Line 145) that previous studies showed that the two clades "reflect independent hybridizations" of lineages A and B seems to be a complete misrepresentation of what those previous papers actually said, which is baffling because two of these papers are from the authors' own lab.

Response: The reviewer is right, and we have now modified this sentence.

Del Olmo et al then go on (Line 159) to identify a third clade (Clade 3) and say that "it likely corresponds to a new hybridization event between A and B", i.e. a third hybridization. But there was no previously-published evidence for more than one A/B hybridization, and Del Olmo et al do not present any convincing evidence for one now. The only thing they present is Figure 2 B,C, which shows that regions of LOH are more strongly conserved within each clade than between clades, but this is not proof that there were multiple independent hybridizations – it is fully consistent with a single shared ancestral A/B hybridization followed by LOH on the branches leading to the Most Recent Common Ancestor of each clade.

Response: We agree with the reviewer and we have now revised this interpretation, leading us to conclude that there is not enough evidence to claim independent hybridization events within A/B hybrids.

One unanswered question is whether the Clade 3 isolates also show the two large blocks of LOH that Mixao et al (2023) commented on. Del Olmo et al don't comment on this.

Another unanswered question is whether Clade 3 shares the LOH at the mating-type (MTL) locus that was seen in Clades 1 and 2. Mixao et al (2023) reported that Clades 1 and 2 have similar patterns of LOH at this locus, with the MTLa idiomorph being partially overwritten by MTLalpha, albeit with slightly different boundaries in the two clades. So does Clade 3 also

have a similar LOH at MTL? If all 3 clades have similar LOHs at MTL, it is an argument for shared ancestry (i.e. a shared single A/B hybridization followed by LOH at MTL in the common ancestor of all 3 clades). The authors' preferred hypothesis of 3 independent A/B hybridizations would imply convergent evolution (3 independent but similar LOHs at MTL) for no obvious reason.

Response: We have now closely investigated the LOH blocks considered in the Mixao et. al. publication. These new results are now included in the revised manuscript. As the reviewer will see, the putative new clade also harbours those blocks, reinforcing the idea that all A/B strains came from a single hybridization event, which was followed by early divergence generating distinct LOH patterns in each sub-clade. We have now revised all relevant parts of the manuscript to reflect that and we thank the reviewer for his/her insights.

The manuscript should be revised to delete the claim that there were multiple A/B hybridizations, and to remove the misrepresentation of previous papers. In C. orthopsilosis, there was clear evidence for multiple independent hybridization events between the same two parental lineages because there were some smoking guns: different hybrid lineages had different MAT genotypes, or different mtDNAs (refs. 23, 28). There is no equivalent smoking gun for multiple A/B hybridizations in C. metapsilosis. Del Olmo et al have done some interesting work, but on the issue of the number of A/B hybridizations they have reached a conclusion that is not supported by the evidence.

Response: See responses above, we have modified this whole part, and agree with the reviewer.

Reviewer #2: The genetic work proposes gene candidates for genetic differences involved in thermotolerance (as well as other phenotypes) and notes that "additional experiments are needed" to link genetic changes in LOH regions to changes in phenotype. There should be a direct experiment in this paper to follow up on one of these candidates and test one or more of the highest confidence predictions and establish LOH as a driver of these phenotypes.

Response: We agree with the reviewer of the suitability of those experiments to reach a final conclusion. However, given the combination of potential related mutations in each strain and the difficulty of precisely engineering an LOH event we consider this experiment out of the scope of this article. We ask the reviewer to consider the fact that the use of CRISPR-Cas in this species is not easy. Given the reports suggesting that CRISPR-Cas induces LOH in the close relative C. parapsilosis (https://www.ncbi.nlm.nih.gov/pmc/articles/PMC9769790/), and considering the higher levels of heterozygosis it is highly likely that similar of-target events will hamper conclusions from genetic manipulation in this system. Therefore at this point we consider direct manipulative experiments to test phenotype-genotype connections in this species are unfeasible. We hope the reviewer agrees with us on this point.

For the correlations the authors draw about drug resistance, these inferences would be stronger if more isolates were measured for sensitivity and for fluconazole across a wider range of drug concentrations. It is unclear why these drug concentrations were selected , particularly the two nearly identical values of fluconazole. There should be more consistency overall across the panels, and replicates in clades or groups (except 4 where that isn't possible) where conclusions are being drawn.

Response: Unfortunately, we do not have in our hands all strains included in the analyses. Many of the data used in this manuscript correspond to publicly available sequences or to strains for which we only obtained DNA due to limitations of obtaining MTA from original sources. We acknowledge that our phenotypic assessment does not cover the whole diversity of each of the tested clades and that some of the differences observed might be strain-specific and not clade-specific, we now have toned down the discussion in this regard. As for the range of concentrations tested, we remind that our purpose is not to determine the MIC but rather to assess differences in growth among strains. We have tested more concentrations and these were the ones that better reflected the range of susceptibilities observed in the tested strains.

The statement of a third hybrisation event is not well supported. The authors need to eliminate the possibility that clade 3 originated from the same hybrisation event and that there were unique LOH events in clade 3. Based on the neighbornet, this looks like a group that is undergoing genetic exchange, so clarifying the level of recombination and the mating types observed may also be helpful.

Response: See our responses above. We have revised this whole part.

Part III – Minor Issues: Editorial and Data Presentation Modifications
Please use this section for editorial suggestions as well as relatively minor modifications of existing data that would enhance clarity.

Reviewer #1: The strength of the claim that there were multiple A/B hybridizations seems to grow as the manuscript progresses. On line 32 “we identify a new clade *possibly* corresponding to an independent hybridization event”. On line 96, “we defined a new hybrid clade that *likely* represents an independent hybridization event”. On line 188, “our analyses have uncovered a new independent clade of hybrids (clade 3) which *corresponds* to a third hybridization event.” This is sloppy use of language – “possibly”, “likely” and “is” all mean different things. In my opinion, the hypothesis that multiple A/B hybridizations occurred remains unproven and no word stronger than “possibly” should be used to describe this hypothesis.

Response: We have modified all text related to the clade discussions

Line 138: Is ref 34 is the right reference here?

Response: Thank you for noting this, we have corrected the citation.

Line 159, Line 379: Clade 3 was also identified and highlighted by O’Brien et al (2022) (Fig 1c, green).

Response: The four strains included in O’Brien et. al. were isolated from two patients from the same hospital. In the referred paper they are depicted in green indicating they come from that study. They are described as clustering together, which is not surprising given their close isolation. However, no claim is made in the paper that this constitutes a new subclade of a similar status to others within the A/B clade. The clade described here is more apparent

both in the SNP-based tree and in the LOH blocks comparison and includes strains from distant geographical locations, which provides evidence that this is a broadly distributed clade, rather than a group of related strains. In the revised manuscript we note that some of the strains formed a distinct cluster in the previous study.

Section beginning on Line 275: Isn't phenotype studied in this section drug resistance, not drug tolerance? See papers by Judy Berman.

Response: We have changed tolerance to susceptibility

Reviewer #2: In the description for Figure 1, panel B is described but not shown.

Response: Panel B was substituted by Supplementary Figure 1. The description of Figure 1 was corrected and references in the text were adjusted.

In Figure 2B, label the isolates that are shown in the plot.

Response: Figure 2 was modified to add the names of the isolates in panel B

What are the two panels in Figure 4 - replication of the same experiment?

Response: Yes, this is now indicated in the legend.

On line 118 the authors note that one isolate is the "first sequenced C. metapsilosis from the African subcontinent". In general, claims of primacy should be discouraged.

Response: We have checked all public sequence repositories. Nevertheless we have added "to the best of our knowledge" to make our claim relative to the publicly available information. We think it is important to note this, as the African continent is usually neglected from studies.

Line 140: The authors need to more precisely describe the genetic relationships of the environmental isolates, such as by number of differences. The phrase "very close" seems misleading looking at the figure, particularly with the very divergent MSK414 included.

Response: This sentence refers to the figure where the distances are shown. We have rephrased the sentence to increase clarity, so that is clear that they are close to some clinical isolates, not to all (e.g. MSK414).

Lines 171-176: how were aneuploidies accounted for in heterozygous variant calling - was there a validation that there was not under-calling in these regions?

Response: We did not do a specific test for this, which is difficult given that we do not have a "control" strain that only differs in the aneuploidy for any given strain.

Lines 310-312: this point about the LOH in ERG11 contributing to tolerance is not strongly supported by the data as only one clade 2 isolate has been measured for drug sensitivity. Additional isolates should be tested to make this point, as there seem to be no strongly supporting variants and a LOH region much larger (?) than the ERG11 locus.

Response: We agree with the reviewer. We now stress that this is merely a hypothesis that needs further support.

The discussion should acknowledge the limitation of looking only at LOH regions for genetic drivers of phenotypes. There are other sources of variation that could contribute.

Response: We have added a sentence in the discussion to stress this.

In the methods for phylogenetic analysis, line 519, there is description of an "in-house script" to create the alignment used for phylogenetic analysis. This should be provided to allow for replication.

Response: The code used for the alignments has been made publicly available at repository: https://github.com/Gabaldonlab/phylogenetic-reconstruction-for-hybrids. A direct link at the Data availability section has been added.

There is a major issue with reference formatting. All of the references after ~reference 34 appear to be mis-numbered and the reference at line 439 has not been formatted.

Response: References have been corrected.

Supp Fig 2 - use consistent clade colors with Fig 2 as it is called out in the text alongside.

Response: Supp Fig 2 has been adjusted to match the colours of Figure 2.

Supp Fig 4 - label what is a statistically significant coverage difference.

Response: Now supplementary Fig 5. We did a visual analysis finding regions with coverage roughly double than the basal coverage, we did not perform a statistical test. We consider the coverage plots show clearly the increase in coverage.

Supp Fig 5 - for PL448, what explains the lack of resolution for scaffold 6?

Response: (now Supp Fig 6) - PL448 had a whole-chromosome LOH in scaffold 6, and the derived low density of SNPs explains the lack of a density peak in that scaffold.

Supp Fig 6 - it would be helpful to group the list of isolates by clade to compare with the main text where is is called out.

Response: (now Supp Fig 7) - Panel B now shows the data grouped and coloured by clade.

PLOS authors have the option to publish the peer review history of their article (what does this mean?). If published, this will include your full peer review and any attached files.

If you choose “no”, your identity will remain anonymous but your review may still be made

public.

Do you want your identity to be public for this peer review? For information about this choice, including consent withdrawal, please see our Privacy Policy.

Reviewer #1: No

Reviewer #2: No

Figure Files:

While revising your submission, please upload your figure files to the Preflight Analysis and Conversion Engine (PACE) digital diagnostic tool, https://pacev2.apexcovantage.com. PACE helps ensure that figures meet PLOS requirements. To use PACE, you must first register as a user. Then, login and navigate to the UPLOAD tab, where you will find detailed instructions on how to use the tool. If you encounter any issues or have any questions when using PACE, please email us at figures@plos.org.

Data Requirements:

Please note that, as a condition of publication, PLOS' data policy requires that you make available all data used to draw the conclusions outlined in your manuscript. Data must be deposited in an appropriate repository, included within the body of the manuscript, or uploaded as supporting information. This includes all numerical values that were used to generate graphs, histograms etc.. For an example see here on PLOS Biology: http://www.plosbiology.org/article/info%3Adoi%2F10.1371%2Fjournal.pbio.1001908#s5.

Reproducibility:

To enhance the reproducibility of your results, we recommend that you deposit your laboratory protocols in protocols.io, where a protocol can be assigned its own identifier (DOI) such that it can be cited independently in the future. Additionally, PLOS ONE offers an option to publish peer-reviewed clinical study protocols. Read more information on sharing protocols at https://plos.org/protocols?utm_medium=editorial-email&utm_source=authorletters&utm_campaign=protocols
