# Supplementary material for: Insights into the origin, hybridisation and adaptation of Candida metapsilosis hybrid pathogens
Source: PLoS Pathog. 2025 Jan 17;21(1):e1012864. doi: 10.1371/journal.ppat.1012864 (PMC11781744; doi:10.1371/journal.ppat.1012864)
Supplement: Attachment — Submitted filename: Comments metapsilosis PLOS pathogens.docx [file ppat.1012864.s015.docx]

Dear Editor,

As you will see we have addressed the remaining minor concern by toning down our hypothesis that LOH is related to differences in susceptibility. We clearly state that it is simply a hypothesis suggested by the data, but that awaits further testing.

Best wishes, Toni

Reviewer's Responses to Questions

Reviewer #2: The correlation of the genomic data with drug resistance still seems very preliminary and over-stated where LOH regions or variants are highlighted that affect genes linked to resistance, however none of the variants appear to have a known association with resistance. Finding novel and LOH variants would be interesting, but the candidates need to be validated; here the numbers aren't large enough to have strong assocications.

**Response:** We have toned down the two sentences where this was stated and we now clearly say it is an speculation that requires further testing.
